# An application of the hybrid AHP-PROMETHEE approach to evaluate the severity of the factors influencing road accidents

**DOI:** 10.1016/j.heliyon.2023.e21187

**Published:** 2023-10-20

**Authors:** Priyank Trivedi, Jiten Shah, Sarbast Moslem, Francesco Pilla

**Affiliations:** a Civil Engineering Department, Institute of Infrastructure Technology Research and Management, [IITRAM], Ahmedabad, India; b School of Architecture Planning and Environmental Policy, University College of Dublin, D04 V1W8, Belfield, Dublin, Ireland

**Keywords:** Severity, Factors influencing road accidents, AHP, PROMETHEE

## Abstract

The evaluation of the severity of the factors influencing road accidents with a detailed severity distribution is critical to plan evidence-based road safety improvements and strategies. However, currently available studies use statistical and machine learning (ML) models to evaluate the severity of factors causing road accidents without a detailed severity distribution. Further, most of these available models require significant pre-data processing and have certain data-centric limitations. However, the multi criteria decision-making (MCDM) techniques have the potential to combine expert opinions for robust analysis without any pre-data processing calculations. Thus, this study uses a hybrid analytic hierarchy process (AHP) and the preference ranking organisation method for enrichment evaluation (PROMETHEE) approach to analyse the severity of factors and characteristics that influence road accidents within the Gujarat state, using injury types as criteria and minor road accident influencing factors as alternatives. These 82 minor factors have been further characterised into 18 characteristics and 4 major factors. Further, AHP integrated 40 expert inputs to determine criterion weights, while PROMETHEE ranked all minor variables. Then, after applying k-mean clustering, each ranked factor has been classified as very severe, moderately severe, or severe. The result clearly highlights that overspeeding, male gender, and clear weather conditions have been concluded to be the highly severe factors for Gujarat state. Thus, by providing a clear severity analysis and distribution of factors influencing road accidents, the proposed research may help government stakeholders, researchers, and politicians build severity-based road safety reforms and strategies with clarity.

## 1 Introduction

Road traffic fatalities and injuries have economic effects such as hospitalization and treatment costs for the victim, health insurance, the diminished value of life, and the victim's family's total output [1]. The majority of young children and adults are killed in traffic accidents [2]. According to the world health organization, road accident fatalities and injuries cost as much as the country's 3–5 % gross national product (GNP) [3]. A detailed global road accident research found that low to middle-income countries (LMICs) are disproportionately affected by deteriorating road safety standards [4]. With a rise in the number of registered motor vehicles, most South Asian nations are seeing an increase in the number of road accidents [5]. India, as a blooming South Asian economy, is also being strongly impacted by the rising issue of traffic accident-related fatalities and injuries. Because of demanding safer road conditions, India recorded 15 road accident deaths and 50 associated injuries each hour [6]. Furthermore, the Indian state-by-state road accident investigation showed that the average number of road accident deaths per 100 incidents increased by 2.3 in 2019 compared to 2016 [7]. According to the Ministry of Road Transport and Highways (MoRTH) of India, road accidents killed 1,51,417 people and injured 4,69,418 people in 2018 [8]. Therefore, the higher numbers of fatalities and injuries caused by road accidents across the India proposes that more realistic approach is required to better examine the factors influencing road accidents. Further, the necessity for supplementary evidence in various contexts is increasingly obvious as a result of insufficient data, such as the relative absence of information on non-fatal accidents, variances in findings, and the definition of accident severity with its associated factors [1]. Thus, it is mandatory to evaluate the severity of factors influencing road accidents by considering fatality and injury numbers. The detailed evaluation of accident-influencing factors may lay the foundation for road safety policies and strategies in the long run. Thus, it is mandatory to evaluate the severity of factors influencing road accidents by considering the fatalities and injury numbers.

Several studies have evaluated the road accident severity and related factors with statistical models and machine learning (ML) algorithms [[9], [10], [11], [12], [13]]. But, most of these studies considered data of fatal accidents and related parameters only. In the same way, the Ministry of Road Transport and Highways (MoRTH) in India uses a metric of deaths per 100 accidents to evaluate the seriousness of traffic incidents. In order to conduct a comprehensive investigation into the severity of road accidents, it is necessary to consider all types of injuries, including those resulting in fatalities. In addition, the existing severity analysis models lack the capability to cluster the road accident influencing factors based on severity. Therefore, present paper aims to provide a novel application of hybrid Multi-Criteria Decision-Making (MCDM) models - the Analytic Hierarchy Process (AHP) and Preference Ranking Organization METHod for Enrichment Evaluation (PROMETHEE) approach to evaluate the severity of road accident influencing factors by considering fatalities, grievous injuries, and minor injuries and to provide a clear severity classification of most factors influencing road accidents.

The paper follows the following arrangement. Section 2 delivers a systematic literature review. Section 3 demonstrates the methodological approach for evaluating the severity of selected accident influencing factors with a hybrid AHP-PROMETHEE approach. Section 4 evaluates and discusses the severity ranking of all 82 factors with proper cluster-wise distribution and graphically representation of the result. Lastly, Section 5 concludes the results of the present research and recommendations.

## 2 Literature review

Many research articles have explored the road accident severity analysis and have proposed solutions to improve the safety of road users [[14], [15], [16], [17]]. The primary focus of most studies pertaining to the severity of road accidents has been the examination of factors linked to fatalities [18,19]. Conversely, there has been comparatively less emphasis retained on grievous and minor injuries [20]. The further detailed investigation of available road severity analysis research article suggests the use of historical accident data [[21], [22], [23]]. But the historical accident data has different issues. The issue such as under-reporting and misreporting of road accident injury data has been consistently emphasized in various countries, thereby hindering the accurate analysis of road accident severity using injury data [24,25]. Numerous scholarly studies have expressed apprehension regarding the inadequate documentation of severe injuries, minor injuries, and non-injuries, which hampers the robust severity analysis [25,26]. The excessive focus given to road accident fatalities in severity analysis leads to the observation that the predominant research emphasis is on mitigating the number of fatalities resulting from road accidents rather than addressing the broader issue of reducing the overall severity of injuries sustained in such accidents. But, it is anticipated to include every type of injury for an accurate severity analysis of factors influencing road accidents [27]. Therefore, the severity of factors influencing road accidents should be analysed with the integration of injury data with fatalities. Sayed et al. [28] had evaluated the accident severity of day and night time with fatalities, grievous injuries and no injuries, only. A few specific studies investigated the severity of different factors on road accidents near intersections with available road accident data [29,30]. But most articles use various statistical methodologies and mathematical models to examine the severity of factors influencing road accidents. The available research work on road accident severity analysis applied complex mathematical models such as binary logit and probit [[31], [32], [33]], multinomial logit [[34], [35], [36]], nested logit [37,38], ordered mixed logit [39], and ordered logit/probit [40,41]. Saha et al. [42] divided these applied models as ordered models and unordered models. However, it should be noted that a majority of these severity models make certain assumptions based on available road accident data [43,44]. Furthermore, the models discussed in this context are subject to certain constraints, including the need for a balanced mean and variance [45,46]. But, contemporary research trends in accident severity analysis emphasise the utilisation of ML techniques with advanced computational power [47]. Some more recent literature applied ML techniques such as Logistic regression, Support Vector Machine (SVM) [48] and Artificial Neural Network (ANN) [49]. The factors influencing road accident severity such as human factors (i.e., gender, age, health condition), roadway factors (i.e., surface condition, type of road) and environmental factors (i.e., time, weather, lighting condition) are being analysed by Classification tree [50], Random forest [51,52], C4.5 algorithm [53], Naïve Bayes [54,55] and k-nearest neighbor [56,57]. But, one noteworthy limitation inherent within ML techniques is their tendency to work as black-box [43], lacking the ability to provide a transparent representation of crash injury severity and the associated explanatory variables. The primary challenge lies in comprehending the outcomes of ML algorithm when applied to the analysis of accident severity [43]. Therefore, it is clear that statistical and ML approaches of road accident severity analysis have some limitations as well as complex calculations.

MCDM models provide a theoretical framework for comprehending intricate research questions [58,59]. MCDM models provides a well-defined structure and computation steps to solve the complex problems of different field such as management research and operational science [60,61], health and medication [62,63], energy sector [64,65], sustainable development [66,67], mobility and urban transport [[68], [69], [70], [71]].

This structure of MCDM employs an arithmetic approach and mathematical computation to assess the performance of various alternatives in relation to specific evaluation criteria. Further, MCDM allows experts inputs to control the problem constrains with limited resource [72]. The expert inputs clarify the varied significance of different criteria present in a MCDM structure and provides a way forward for computing criteria weights. While examining various criteria for weighting computational methods, the Analytic Hierarchy Process (AHP) emerges as the predominant technique for determining the weights of these criteria through pair-wise comparisons [73,74]. The study aims and the characteristics of the decision-making process determine how the AHP evaluates various criteria by breaking them down into a hierarchical structure. Consequently, this phenomenon has the effect of reducing cognitive errors and potentially confirming the decision-makers' coherence regarding prioritization [75]. Furthermore, the unique structure of AHP is capable of assessing both quantitative and qualitative factors of selected evaluation criteria [76]. Hence, the Analytic Hierarchy Process (AHP) is frequently employed as a systematic and comprehensive methodology for determining the weights of criteria while operating within specified limitations of time and resources. Many researchers solved very complex problems of industries with the AHP such as Durmuşoğlu evaluated different techno-entrepreneurship ventures with AHP [77], Petruni et al. [76] analysed the reliability evaluation techniques of human for the automotive industry, Nikkah et al. [78] developed AHP-based weighting framework for life cycle assessment of agro products. Further, the available literature highlights the application of AHP within transportation sectors such as service development for public transport [72], sustainable urban mobility and planning [79], urban transport development [80], green transport planning [81], last-mile delivery [82], and rail transport [83]. The widely recognised techniques of Multiple Criteria Decision Making (MCDM) combined with Analytic Hierarchy Process (AHP) involve a wide range of approaches, including, the Best-Worst Method (BWM) [84], the Complex Proportional Assessment (COPRAS) [84], the Evaluation Based On Distance From Average Solution (EDAS) [85], the Multi-Attributive Border Approximation Area Comparison (MABAC) [86], the Multi-Attribute Ideal-Real Comparative Analysis (MAIRCA) [87], Multi-Objective Optimisation by a Ratio Analysis plus the Full Multiplicative Form (MULTIMOORA) [88], the Tomada de Decisao Interativa Multicriterio (TODIM) [89], the Technique for Order Preference by Similarity to Ideal Solution (TOPSIS) [90], Vlse Kriterijumska Optimizacija Kompromisno Resenje (VIKOR) [91], the Weighted Aggregated Sum Product Assessment (WASPAS) [92], and the Preference Ranking Organisation METHod for Enrichment Evaluation (PROMETHEE) [93]. However, the PROMETHEE properly displays outranking outcomes of alternatives by employing several preference functions [94]. The PROMETHEE method is regarded as relatively simpler in terms of both computation and conceptualization when compared to other Multiple Criteria Decision Making (MCDM) methods [95]. Ulengin et al. defined the PROMETHEE as a user-friendly, outranking MCDM method with the potential to solve complex problems with higher accuracy [96]. Significantly, the integrated framework of the AHP and PROMETHEE methodologies effectively addresses complex decision-making processes and challenges related to ranking alternatives [97]. Various methodologies have been employed in scholarly literature to address transport and mobility challenges. These include the application of AHP-PROMETHEE-based policy evaluation factors to assess the viability of clear fleet initiatives [98], the examination of freight villages to analyse their impact on transportation [99], the evaluation of public transport modes to assess their quality [93], the selection of bus chassis for larger fleet operations [100], and analysis of urban transport modes with different service quality alternatives [101]. But there is a clear lack of application of the hybrid AHP-PROMETHEE-based modelling approach to evaluate the severity of factors influencing road accidents.

The comprehensive literature review emphasises that both traditional statistical models and contemporary machine learning algorithms for evaluating the severity of factors impacting road accidents possess certain limitations. Moreover, existing scholarly literature has employed historical accident datasets that possess a restricted number of accident-influencing factors and subsequently derived severity distributions using different clustering techniques [14]. Further, none of the currently available severity evaluation models and algorithms have integrated expert inputs due to their data-centric nature. Despite possessing sufficient potential to solve the most complex problems, the potential of MCDM techniques like AHP and PROMETHEE remains untapped in the assessment of the severity of factors influencing road accidents. As a result, the current study aims to apply an AHP-PROMETHEE hybrid approach to investigate and conclude the severity of road accidents influencing factors.

Based on the research gaps identified at the conclusion of a comprehensive literature review, this study makes the following original contributions:

I. The present study represents a novel application of a hybrid AHP-PROMETHEE approach to evaluate the severity of road accident influencing factors by combining fatalities, grievous injuries, and minor injuries.
II. The present methodology provides a way to incorporate the experts input for severity analysis.
III. The study delivers a distinct way to provide severity ranks with a severity distribution of factors influencing road accidents. Further, a unique graphical representation of this severity distribution has been included in the present study.
IV. The proposed approach does not need any pre-data processing or data cleaning steps.

## 3 Data and methodology

**Fig. 1:**
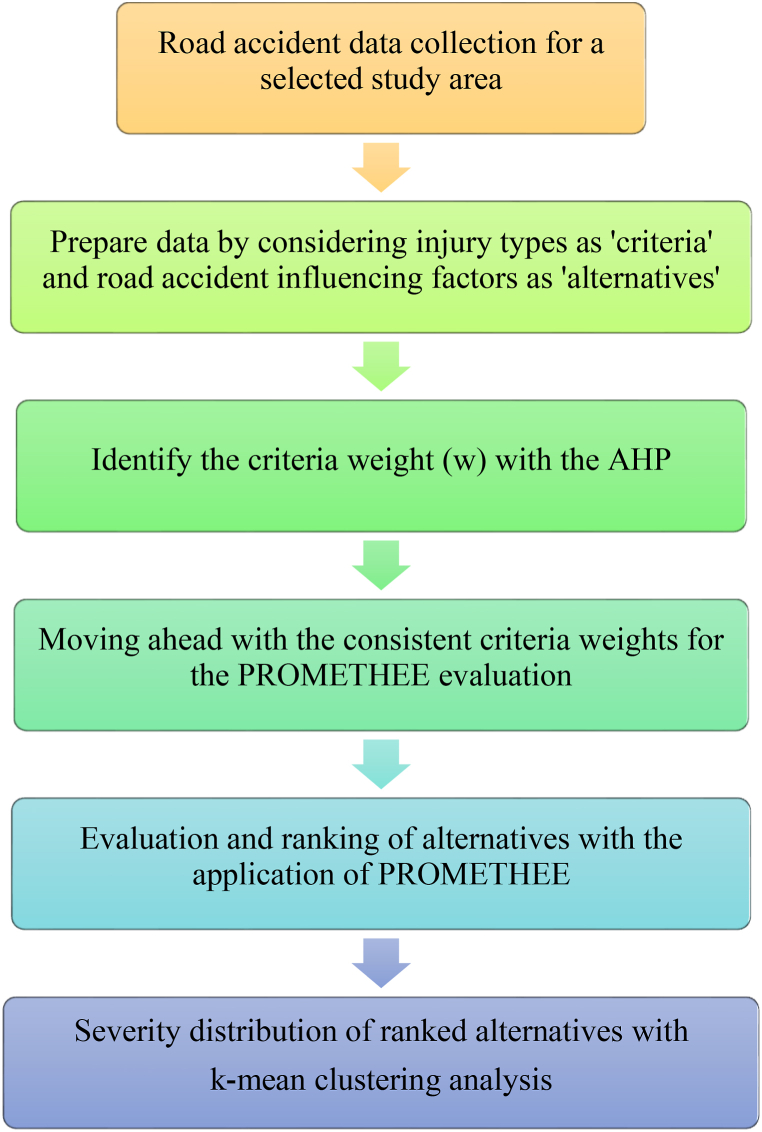
Flowchart of the proposed methodology.

The road accident data collection is a crucial component of the present study as it governs the accuracy of severity analysis and distribution. To attain the aim of study, road accident data for Gujarat state is considered. Gujarat state is dealing with escalating road safety difficulties, placing seventh in the Indian state-by-state road accident severity ranking [20]. Gujarat state administrative authorities are attempting to improve road safety by launching safety campaigns and public awareness campaigns. However, evident-centric research is still required to identify the causes related to road accident fatalities and injuries. Therefore, the road accident data for year 2021 has been collected from the Gujarat Road Safety Authority (GujROSA). According to the information presented in Table 1, the collected data has been organised into various 'alternatives' and ‘criteria’. In order to conduct more comprehensive investigations, the injury data are categorized into various injury types, namely fatalities, grievous injuries, and minor injuries, which are referred to as ‘criteria’. Additionally, there are 82 minor factors that are considered 'alternatives' and play a role in influencing the severity of road accidents. These factors are taken into account to facilitate more detailed analyses. Fig. 1 represents the flowchart of the proposed methodology.

**Table 1:** Fatality and injury data classified by characteristics and minor factors for Gujarat state in 2021.

Characteristics	Minor factors/Alternatives (An)	Fatalities (C1)	Grievous Injuries (C2)	Minor Injuries (C3)
Weather Conditions	Sunny/Clear (A1)	6322	6730	4927
	Rainy (A2)	553	628	588
	Foggy & Misty (A3)	284	215	180
	Hail/Sleet (A4)	7	4	0
Road Type	Expressways (A5)	52	57	33
	National Highways (A6)	2025	1507	1184
	State Highways (A7)	2391	2556	1794
Area Type	Residential (A8)	1170	1388	1062
	Institutional (A9)	472	497	417
	Commercial (A10)	703	951	727
	Open Area (A11)	4976	4792	3537
Road Features	Straight Road (A12)	6073	6318	4522
	Curved Road (A13)	727	781	914
	Bridge (A14)	261	255	165
	Culvert (A15)	49	45	22
	Pot Holes (A16)	5	4	4
	Steep Grade (A17)	71	76	51
	Ongoing road works/Under construction (A18)	154	190	131
Junction Type	T Junction (A19)	366	484	364
	Y Junction (A20)	161	205	141
	Four Arm (A21)	388	467	400
	Staggered (A22)	619	653	367
	Round About (A23)	142	206	160
Traffic Control	Traffic light Signal (A24)	50	97	66
	Police controlled (A25)	87	89	90
	Stop sign (A26)	34	46	26
	Flashing signal/blinker (A27)	8	11	12
	Uncontrolled (A28)	1497	1772	1238
Pedestrian infrastructure	Zebra crossing (A29)	139	140	97
	Foot bridge/Subway (A30)	7	9	5
	Footpath (A31)	71	63	34
	No pedestrian infrastructure (A32)	1256	763	353
Vehicle type	Pedestrian (A33)	1473	975	489
	Bicycles (A34)	111	125	58
	Two wheelers (A35)	3522	3866	2528
	Auto rickshaws (A36)	376	642	529
	Cars, Taxis, Vans & LMV (A37)	939	1305	1264
	Trucks/Lorries (A38)	570	510	493
	Buses (A39)	79	175	269
	Other non-motorized vehicle (E-rickshaw etc.) (A40)	42	54	44
Vehicle age	Less than 5 years (A41)	2207	2551	2166
	5 — 10 years (A42)	2237	2550	1948
	10.1–15 years (A43)	1230	1327	910
	> 15 years(A44)	650	920	498
Vehicle Loading conditions	Normally loaded (A45)	4026	4808	3524
	Overloaded/hanging (A46)	361	445	369
	Empty (A47)	1937	2095	1629
Collision type	Hit & Run (A48)	1204	531	314
	Hit with parked vehicle(A49)	193	264	261
	Hit from back (A50)	1873	2331	1815
	Hit from side (A51)	1424	1878	1332
	Run off road (A52)	752	885	540
	Hit with fixed object (A53)	274	255	209
	Vehicle overturn (A54)	584	537	526
	Head on collision (A55)	979	1041	765
Traffic Violation	Over-Speeding (A56)	7168	7298	5317
	Drunken driving/Consumption of alcohol & drug (A57)	15	27	30
	Driving on wrong side (A58)	203	396	327
	Jumping red light (A59)	1	1	4
	Use of mobile phone (A60)	37	73	132
No use of Safety device	Drivers not wearing helmets (A61)	1947	1486	765
	Passengers not wearing helmets (A62)	719	820	595
	Drivers not wearing seatbelts (A63)	399	391	212
	Passengers not wearing seatbelts (A64)	313	404	376
Time	6:00 to 9:00 (DAY) (A65)	797	897	613
	9:00 to 12:00 (A66)	1006	1242	892
	12:00 to 15:00 (A67)	1098	1274	943
	15:00 to 18:00 (A68)	1201	1444	1058
	18:00 to 21:00 (A69)	1663	1577	1100
	21:00 to 24:00 (A70)	880	764	532
	00:00 to 3:00 (A71)	460	332	370
	3:00 to 6:00 (A72)	338	311	306
Localities	Urban (A73)	2061	2783	1902
	Rural (A74)	5391	5066	3939
Gender	Male (A75)	6473	6468	4556
	Female (A76)	979	1381	1285
Driving role	Drivers (A77)	3573	3599	2298
	Pedestrian & Passengers (A78)	3879	4250	3543
Climatic seasons	Summer (A79)	1787	1715	1265
	Monsoon (A80)	2300	2542	1976
	Post-monsoon (A81)	1357	1395	1058
	Winter (A82)	2008	2191	1542

As per the subsequent section, the AHP was employed to assess the weights of the criteria. This utilisation of the AHP framework enables the assessment of the coherence of criteria weights through the incorporation of expert opinions alongside extensive datasets. In addition, the obtained criteria weight is integrated with the PROMETHEE technique in order to accomplish the research aim. Further with the k-mean clustering technique, the PROMETHEE result was grouped into three cluster (i.e, highly severe, moderately severe, severe).

### 3.1 Analytic hierarchy process (AHP)

AHP provides a straightforward decision-making structure as well as precise criterion weights based on expert input. In addition to these benefits, AHP is simple to implement and comprehend. As a result, this method is becoming popular and broadly applicable. The approach is comprised of three key steps:

#### 3.1.1 Decision hierarchy construction

For each multi-criteria decision-making problem, it is critical to create a decision hierarchy. This hierarchy computes the weight of criteria from gathered possibilities based on the decision-making goal. Many complex circumstances need the usage of a multi-level hierarchy with sub-objectives and sub-criteria.

#### 3.1.2 Setting up the comparative importance of criteria

The expert's inputs are collected to establish the comparative importance of every decision-making criteria. The importance score inputs from every expert are collected via interview or online questioner. The definition of this importance score system is provided by Saaty's nine-point importance scale as per Table 2 [102].

(1) $$A=\left[\begin{array}{ccc} 1 & a1_{2} & \begin{array}{cc} \ldots & a1_{h} \end{array}\\ a21 & 1 & \begin{array}{cc} \ldots & a2_{h} \end{array}\\ \begin{array}{c} \vdots \\ ah_{1} \end{array} & \begin{array}{c} \vdots \\ ah_{2} \end{array} & \begin{array}{cc} \begin{array}{c} \ddots \\ \ldots \end{array} & \begin{array}{c} \vdots \\ 1 \end{array} \end{array} \end{array}\right],\left(i,j=1,2,.\;.\;.,h\right)$$

**Table 2:** Saaty's nine-point importance scale [102].

Importance score	Definition
1	Equal importance
2	Weak importance
3	Moderate importance
4	Moderate and above importance
5	Strong importance
6	Strong and above importance
7	Very strong importance
8	Very, very strong importance
9	Extreme importance

Considering the criteria set as C = {C_j_ ǀ j = 1, 2, . …, h}. The findings of the comparative pairwise analysis of *h* criteria are summarized in the assessment of matrix A. The quotient of criteria weights as a_ij_ is represented by this (*h* × *h*) ordered matrix as per equation (1).

According to equation (2), the right eigenvector (w) and the greatest eigenvector (max) determine the relative priority.

(2) $$A\;w=\lambda_{\max}\;w$$

The evaluation matrix A must have rank 1 for each consistent pairwise comparison condition, and the greatest eigenvector (*λ*_max_) must become equal to the number of criteria (h).

Every subsystem in the hierarchy adheres to the aforementioned procedure. To determine the priority vectors, the vectors must be weighted by the global priority, beginning at the top of the hierarchy. Here, the relative ranking of options reflects their contribution to the problem statement from the standpoint of the stakeholders [103].

#### 3.1.3 Checking consistency

For entries of the matrix, A: a_ij_. a_jk_ = a_ik_ defines consistency. Further, equation (3) indicated the Consistency Index (CI).

(3) $$CI=\frac{\left(\lambda_{\max}-h\right)}{\left(h-1\right)}$$

Equation (4) leads to a conclusion on consistency based on the Random Index (RI) and Consistency Index values (CI). Table 3 represents the values of RI for the number of criteria (h). The ultimate consistency of expert inputs and decision hierarchy is determined by the consistency ratio (CR). The value of CR must be less than 0.1 for a consistent outcome. The estimated weights of the criterion are acceptable for a consistent outcome.

(4) $$CR=\frac{CI}{RI}$$

**Table 3:** Random Index (RI) values [104].

*h*	1	2	3	4	5	6	7	8	9
RI	0	0	0.58	0.90	1.12	1.24	1.32	1.41	1.45

### 3.2 Preference ranking organization method for enrichment evaluation (PROMETHEE)

Brans et al. [94] advanced PROMETHEE outranking technique with a simple theory. As per equation (5), this method solves the MCDM problem with a finite number of alternatives and criteria [94].

(5) $$\max\;\left\{f_{1}\left(a\right),.\;.\;.,f_{h}\left(a\right)\;ǀ\;a\in A\right\}$$

Here, *f*_j_ are evaluation functions of alternatives that need to be maximized for *h* criteria.

The preference function (P) of two alternatives a, b ∈ A; needs to be identified while comparing them with each other as per equations (6), (7)).

(6) P(a,b)=F(d)=F[f(a)−f(b)]

(7) 0≤P(a,b)≤1

Here, P (a, b) is the preference function of criteria a and b; f (d) is the function increasing deviation (d) among f (a) and f (b).

Decision-makers must choose from six preference function types with any two threshold parameters represents (p, q, or s) [105]. Table 4 represents these six preference functions. Frequently, the decision-maker disregards the largest divergence, also known as the indifference threshold (q). Nevertheless, the complete preference function follows the lowest deviance, known as the preference threshold (p).

**Table 4:**
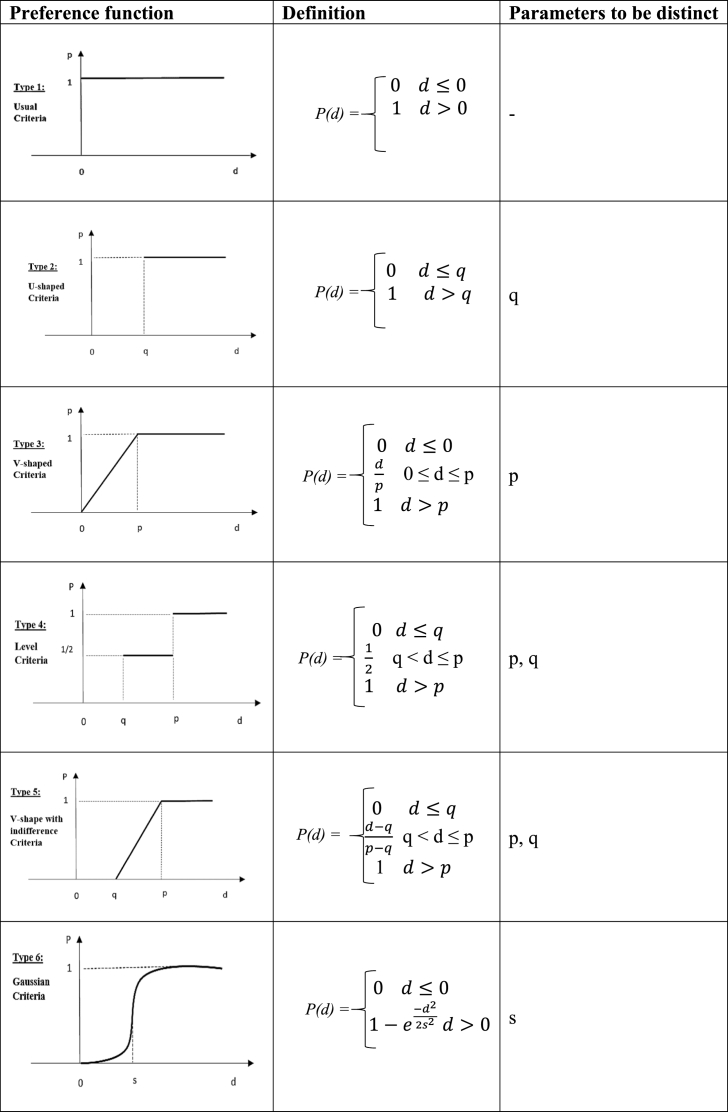
Types of Preference Functions [106]

Further, the PROMETHEE method evaluates succeeding terms for a and b alternatives:

$$\pi \left(a,b\right)=\frac{\sum_{j=1}^{h}w_{j}p_{j}\left(a,b\right)}{\sum_{j=1}^{h}w_{j}}$$

$$\varphi^{+}\left(a\right)=\sum_{b\epsilon A}\pi \left(a,b\right)$$

$$\varphi^{-}\left(a\right)=\sum_{b\epsilon A}\pi \left(b,a\right)$$

(8) $$\varphi \left(a\right)=\varphi^{+}\left(a\right)-\varphi^{-}\left(a\right)$$

Where w_j_ represents criteria weight.

Equation (8) calculates the total preference index of alternatives ‘a’ over alternative ‘b’ using preference indices π (a, b). Furthermore, the leaving flow (φ^+^(a)) computes the superiority of alternative ‘a’ over others. Similarly, the entering flow (φ^−^(a)) shows the features of the remaining alternatives that outrank choice ‘a.’ Finally, the netflow (φ (a)) reflects alternative as dominance by considering the leaving flow and the entering flow as per equation (8). The PROMETHEE I ranks the alternatives by acknowledging both the leaving and entering flows, whereas the PROMETHEE II primarily considers net flow values. Furthermore, the GAIA graphically represents this final ranking to aid in decision-making [94].

## 4 Results and analysis

**Fig. 2:**
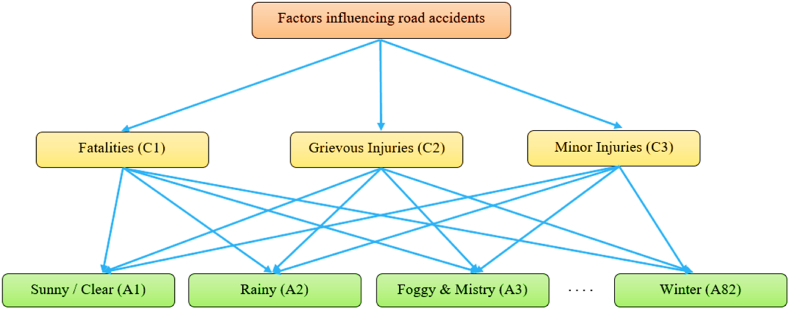
Structure of decision-making hierarchy.

Fig. 2 represents the decision-making hierarchy structure, which combines three criteria on the second level and 82 alternatives on the third level. The hierarchy examines the severity of every road influencing factor (i.e., alternatives) with every possible injury type (i.e., criteria). As per the AHP methodology, a digital survey was distributed to a group of 40 experts in order to ascertain the relative significance of each criterion through pairwise comparisons. Then, the matrix for pairwise criterion comparison is subsequently calculated using the average importance score values obtained from surveys (Table 5).

**Table 5:** Matrix of pairwise criteria comparison.

Criteria	C1	C2	C3
**C1**	1	5	6
**C2**	1/5	1	3
**C3**	1/6	1/3	1

According to the AHP methodology, the following values determined the consistency of criterion weights.

***λ***_max_ = 3.09

RI = 0.58

CR = 0.081 < 0.1

*w* = (0.71, 0.20, 0.09).

The calculated criteria weights (w) exhibit a consistency ratio (CR) below the threshold of 0.1. Therefore, these criteria were applied within the further evaluation process.

Here, the fatalities (C1) resulted in the highest criteria weight values (e.g., 0.71). Subsequently, the grievous injuries (C2) and minor injuries (C3) were assigned criteria weight values of 0.20 and 0.09, respectively. A greater weight assigned to a specific criterion signifies a greater relative significance of a particular injury type (e.g., fatality) in comparison to others. This implies that the utmost importance is placed on fatalities in comparison to grievous and minor injuries. Moreover, it is important to note that all of the criteria weights possess non-zero values. Hence, the weight values derived from expert opinions suggest that the inclusion of all types of injuries is crucial in assessing the overall severity of an accident and any factors that may contribute to road accidents.

Now to evaluate the alternatives with PROMETHEE analysis, the V-shape preference function has been selected for the present study with the help of the VISUAL PROMETHEE software's preference function aid. As per Table 6, each of the 82 alternatives has been put together into 18 characteristics. Finally, these 18 characteristics have been further grouped into 4 major factors, which include human, environmental, road, and vehicle factors. According to the data presented in Table 6, each of the four major factors (e.g., human, environmental, etc.) has been distinctly associated with a particular geometric shape (e.g., circle, square, etc.), while the characteristics (e.g., gender, driving role, etc.) have been assigned individual colours (e.g., white, fuchsia, etc.). This distinctive illustration offers a detailed visual representation of the outcomes obtained from the PROMETHEE analysis.

**Table 6:** Representation of major factors, characteristics, and minor factors in VISUAL PROMETHEE.

Major factors	Shape	Characteristics	Color	Minor factors (Alternatives)
Human factors	Circle	Gender	White	• Male (A75), • Female (A76)
		Driving role	Fuchsia	• Drivers (A77), • Pedestrian & Passengers (A78)
		No use of safety device	Aqua	• Drivers not wearing helmets (A61), • Passengers not wearing helmets (A62), • Drivers not wearing seatbelts (A63), • Passengers not wearing seatbelts (A64)
		Traffic violation	Lime	• Over -Speeding (A56), • Drunken driving/Consumption of alcohol & drug (A57), • Driving on wrong side (A58), • Jumping red light (A59), • Use of mobile phone (A60)
Environmental factors	Square	Weather conditions	Blue	• Sunny/Clear (A1), • Rainy (A2), • Foggy & Misty (A3), • Hail/Sleet (A4)
		Time	Silver	• 6:00 to 9:00 (DAY) (A65), • 9:00 to 12:00 (A66), • 12:00 to 15:00 (A67), • 15:00 to 18:00 (A68), • 18:00 to 21:00 (A69), • 21:00 to 24:00 (A70), • 00:00 to 3:00 (A71), • 3:00 to 6:00 (A72)
		Climatic seasons	Olive	• Summer (A79), • Monsoon (A80), • Post -monsoon (A81), • Winter (A82)
Road factors	Square with Fuchsia colored outline	Road type	Teal	• Expressways (A5), • National Highways (A6), • State Highways (A7)
		Area type	Red	• Residential (A8), • Institutional (A9), • Commercial (A10), • Open Area (A11)
		Locality	Yellow	• Urban (A73), • Rural (A74)
		Road features	Maroon	• Strait road (A12) • Curved road (A13) • Bridge (A14), • Culvert (A15), • Pot Holes (A16), • Steep Grade (A17), • Ongoing road works/Under construction (A18)
		Junction type	Fuchsia	• T Junction (A19), • Y Junction (A20), • Four Arm (A21), • Staggered (A22), • Round About (A23)
		Traffic control	Navy	• Traffic light Signal (A24), • Police Controlled (A25), • Stop Sign (A26), • Flashing signal/blinker (A27), • Uncontrolled (A28)
		Pedestrian infrastructure	White	• Zebra crossing (A29), • Foot bridge/Subway (A30), • Footpath (A31), • No pedestrian infrastructure (A32)
Vehicle factors	Diamond	Vehicle type	Aqua	• Pedestrian (A33), • Bicycles (A34), • Two wheelers (A35), • Auto rickshaws (A36), • Cars, Taxis, Vans & LMV (A37), • Trucks/Lorries (A38), • Buses (A39), • Other non - motorized vehicle (E -rickshaw etc.) (A40)
		Vehicle age	Blue	• Less than 5 years (A41), • 5–10 years (A42), • 10.1–15 years (A43), • > 15 years (A44)
		Vehicle loading condition	Silver	• Normally loaded (A45), • Overloaded/hanging (A46), • Empty (A47)
		Collision type	Red	• Hit & Run (A48), • Hit with parked vehicle (A49), • Hit from back (A50), • Hit from side (A51), • Run off road (A52), • Hit with fixed object (A53), • Vehicle overturn (A54), • Head on collision (A55)

**Fig. 3:**
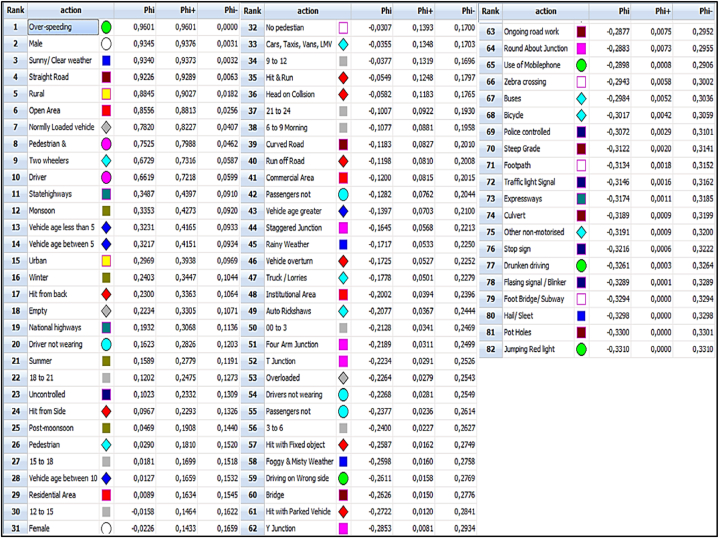
PROMETHEE flow table output from Visual PROMETHEE software.

The PROMETHEE model employs criteria weights generated by the Analytic Hierarchy Process (AHP) to prioritise the alternatives according to the prescribed methodology. The establishment of cost and benefit criteria is a crucial component in the implementation of the PROMETHEE model [107]. In order to accurately assess the severity of each alternative, it is imperative to maximise all three criteria outlined in this specific study. Hence, all three criteria have been taken into account as the benefit criteria for assessing severity. As indicated in the methodology section, the PROMETHEE I method was utilised to rank the alternatives by taking into account both the entering flow (φ^−^(a)) and the leaving flow (φ^+^(a)). In contrast, PROMETHEE II evaluates the net flow, denoted as phi (φ^−^(a)), and subsequently assigns a ranking to each alternative. The resulted numerical values of phi-, phi+, and phi for each alternative are summarized within Fig. 3. The overspeeding (A56) emerged as the top-ranked alternative, exhibiting the highest phi value. Remarkably, the same alternative was assigned the highest rank due to its possession of the lowest phi-value and the highest phi + value. Therefore, the results obtained from PROMETHEE I and II models unequivocally demonstrate that overspeeding was ranked as the top road accident influencing factor. Similarly, a detailed analysis of the rest of the alternatives highlighted that the rankings resulted from PROMETHEE I and II were similar. Further, Fig. 3 uniquely represents every alternative rank with a geometric shape and color mentioned in Table 6.

**Fig. 4:**
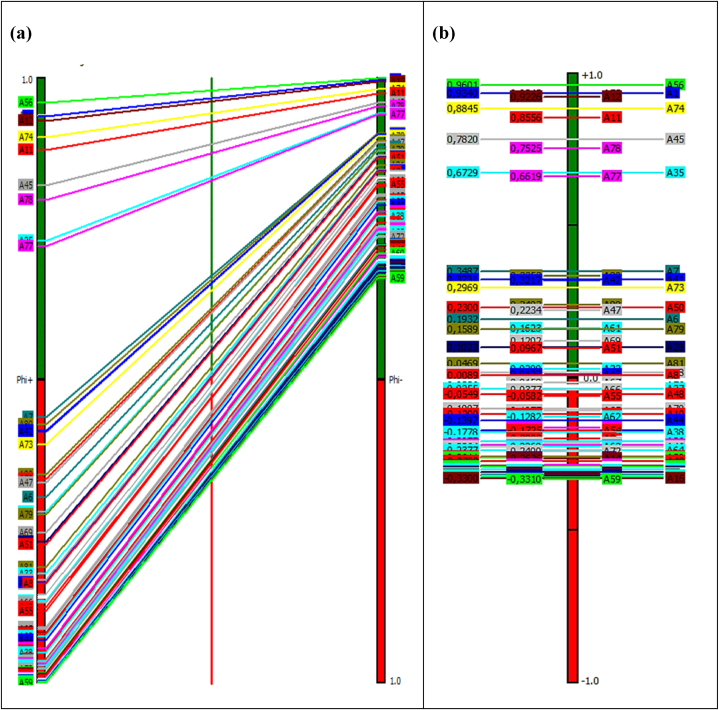
(a) PROMETHEE I ranking (b) PROMETHEE II ranking.

Based on the given fact, Fig. 4 (a) and (b) illustrate the graphical representations of the rankings obtained from PROMETHEE I and II, respectively for better analysis. Here, it is important to note that Visual PROMETHEE software uses a comma () as a decimal separator.

Fig. 4 (a) clearly indicates the PROMETHEE I ranking of every alternative with a phi^−^ and phi^+^ value in either column. The nature of the PROMETHEE I ranking is represented by the lines linking these columns. A line on top of another line represents a preference for one alternative over another. The intersection of these lines denotes the incomparability or conflicting ranking of an alternative with phi^−^ and phi^+^ values. It is interesting to note that none of these lines intersect with each other in Fig. 4 (a). Further, overspeeding (A56) emerged as the top-ranked alternative by phi^−^ and phi^+^ values. Fig. 4 (a) also represents the similar ranking similarity of every alternative with phi^−^ and phi^+^ values. Similarly, Fig. 4 (b) represents that overspeeding (A56) is the first-ranked alternative with the highest phi value. Finally, while analysing the ranking provided by Fig. 4 (a) and (b), the total ranking similarity for every alternative can be easily observed. Moreover, the outcomes of the PROMETHEE I and II rankings signify the distinctive outranking characteristics. This implies that the assigned severity ranking of all 82 factors influencing road accidents signifies their absolute superiority over one another. The higher-ranked alternative exhibits greater severity compared to the lower-ranked alternative. Hence, among the 81 factors examined in this study, the one with the lowest severity is the act of jumping a red light (A59).

**Fig. 5:**
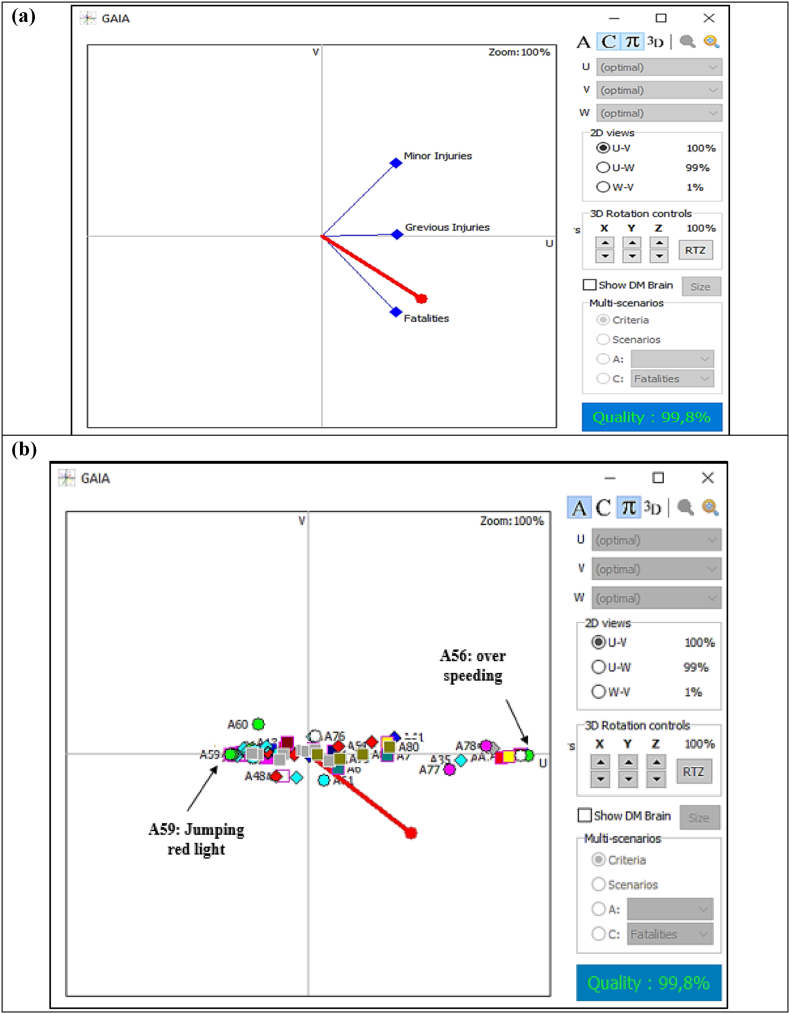
(a) GAIA plane representation of criteria (fatalities, grievous injuries, minor injuries). (b) GAIA plane representation of alternatives (accident influencing factors).

Now after getting the ranking from the PROMETHEE I and II, the geometrical analysis for interactive aid (GAIA) analysis has been conducted within visual PROMETHEE software. The GAIA plane concludes a prospect of possible interaction between research alternatives and criteria [95]. The GAIA analysis sets criteria and alternatives inside several planes (e.g., U–V, U–W, W–V) and represents a graphical representation to facilitate improved decision-making. The arrangement of criteria and alternatives in a similar direction within the GAIA plan represents the identical maximizing or minimizing characteristic [105]. Fig. 5 (a) and (b) illustrates the GAIA plane representation for all three criteria and 82 alternatives, individually. The red decision axis illustrates the decision-makers’ brains for this research. This axis often refers as the decision axis too.

Fig. 5 (a) represents all the criteria axes in an approximately similar direction. This indicates that all three criteria—fatalities, serious injuries, and minor injuries—express similar preferences over all the alternatives. The longer length of the decision axis (π) concludes the very good destiny-driven decision-making nature of the present study. Further, The quality number indicates that 99.8% of the material has been compiled and settled, and none of the alternatives or criteria information is missing from the GAIA representation. Further detailed analysis indicates that the alternatives with a positive net flow are located on the right side of the V-axis, whereas alternatives with a negative net flow are located on the left side (Fig. 5(b)). Therefore, Fig. 5 (b) represents the first 29 ranked alternatives on the right side of the V-axis and the rest of the alternatives on the left side. This representation indicates the higher severity influence of the first 29 ranked alternatives. This further means that moving from right to left on the U-axis, the rankings of the alternatives decrease. Therefore, the right-most lime circle in Fig. 5 (b) represents the overspeeding (A56), while the left-most lime circle represents the jumping the red-light (A59). Therefore, the GAIA representation of the present research provides a one-of-a-kind representation of the severity analysis of factors influencing road accidents. But changes in criteria weights may influence changes in the current ranking of alternatives and current GAIA representation. Hence, a sensitivity analysis has been performed to identify the stability intervals of the criteria weights.

Triantaphyllou and Sánchez [108] observed a relative change in the ranking of alternatives with the modification of criteria weights. This modification also affected the GAIA representation and overall decision-making evaluation. For most decision-making problems, it is important to do a sensitivity analysis to find the weight stability interval (WSI), which is the range of weight changes that don't change the order of the alternative ranking [109]. Thus, the sensitivity analysis for the present research has been conducted according to Table 7. The analysis revealed the WSI for fatalities (C1), severe injuries (C2), and minor injuries (C3). There are no criteria with a minimum or maximum WSI value of 0 or 1. This demonstrates that the ranking of the factors impacting road accidents is governed by all three injury types, accordingly. Further analysis of WSI values of every injury type states that the minimum and maximum values of every criteria weights are value close to computed criteria weights (Table 7). This indicates that the minor change in the weight of any injury type will change the concluded severity raking of factors influencing the road accidents.

**Table 7:** Weight Stability Interval of every criteria.

Criteria	Weight	Weight Stability Interval	
		Minimum weight value	Maximum weight value
Fatalities (C1)	0.71	0.70	0.72
Grievous Injuries (C2)	0.20	0.17	0.20
Minor Injuries (C3)	0.09	0.08	0.09

Now that the WSI values have been concluded, the closing analysis for the PROMETHEE ranking has been conducted. It has been observed that the PROMETHEE model incorporates all three injury types very well to determine the severity rank of factors influencing road accidents. Overspeeding (A56) is a top-ranked minor factor according to the PROMETHEE I and II ranking and GAIA plane analysis. Consequently, minor factors such as male (A75), sunny or clear weather (A1), straight road (A12), rural area (A74), open area (A11), normally loaded vehicle (A45), pedestrians and passengers (A78), two-wheelers (A35), and driver (A77) have been ranked among the top ten severe factors influencing road accidents. These top-ranked factors represent all four key accident-influencing categories (i.e., human factors, environmental factors, road factors, and vehicle factors). This ranking represents the severity order of every factor influencing road accidents within Gujarat state. Further analysis has been conducted to classify these 82 road accident influencing factors into a particular number of clusters for more descriptive severity analysis. Typically, cluster-wise classification produces more descriptive results than ranking data points [110]. Therefore, this research further clustered the PROMETHEE II-ranked alternatives into a particular number of clusters based on the net flow values to determine the severity level.

**Fig. 6:**
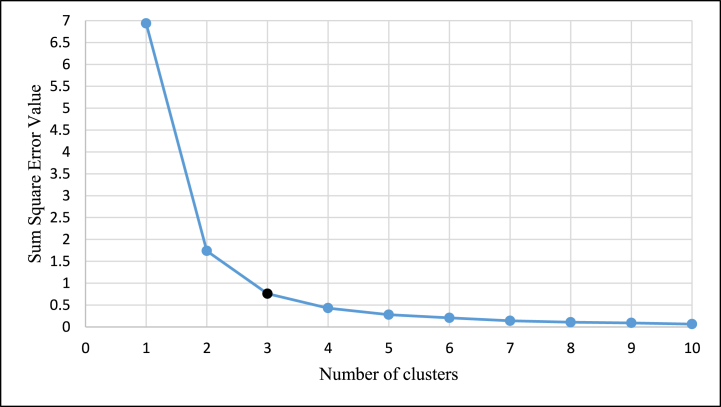
Elbow curve.

The majority of available clustering algorithms group data based on predetermined cluster numbers [111]. But it is very essential to determine the optimal number of clusters within which the overall ranking or data points might be classified. Numerous studies have determined that the elbow curve is the most effective technique for determining the ideal number of clusters [[112], [113], [114]]. According to the elbow method, the sum of square error (SSE) values fall linearly after the elbow point. And the elbow point represents the optimal number of clusters. Therefore, the present research applied the elbow curve method and identified the SSE values for several numbers of clusters as per Fig. 6. The resulting curve demonstrates that the SSE values decrease linearly after three cluster numbers. Further, there is no indication of a rise in SSE values beyond three cluster numbers. As a result, the black highlighted point is considered to be the elbow point, and the 82 accident-influencing minor elements are divided into three groups (Fig. 6).

**Fig. 7:**
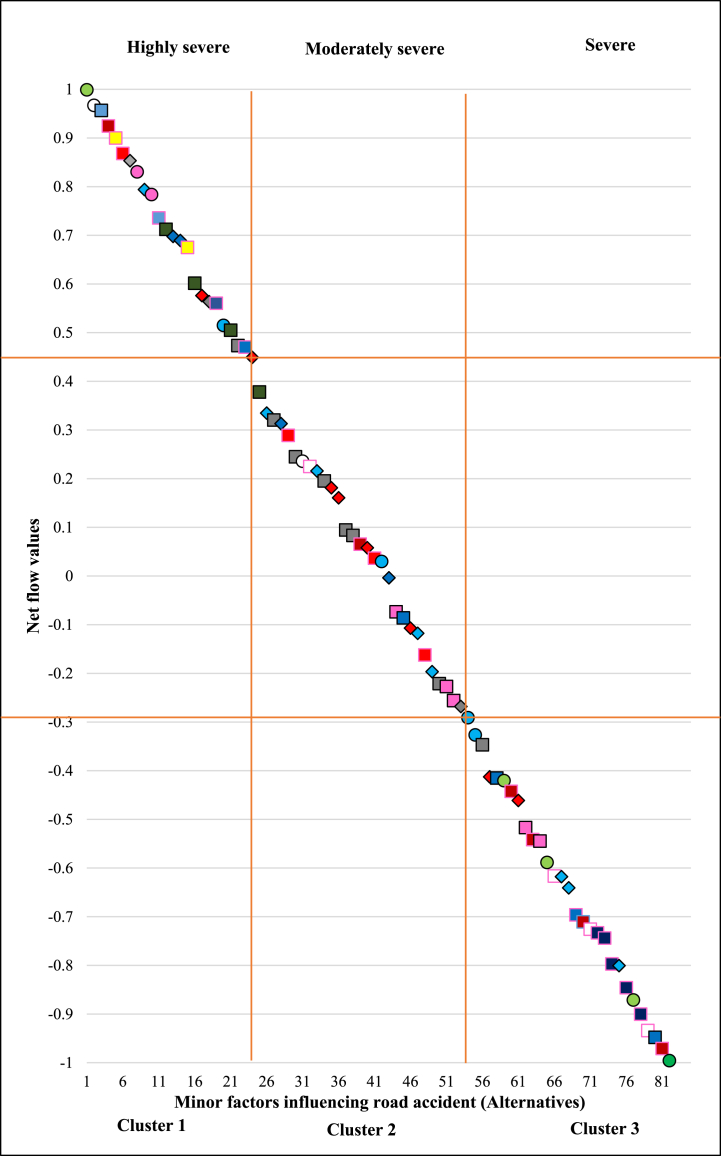
Cluster-wise plot of minor factors influencing road accidents (alternatives).

Now, the selection of clustering algorithms becomes crucial after deciding the number of clusters. Among many available clustering techniques and algorithms, The K-mean clustering technique is popular because it yields accurate classification results in a short amount of time by skipping several iterations [115]. This approach classifies data points into clusters without requiring any data training [116]. As a result, the present research has utilised the k-mean clustering technique to cluster the PROMETHEE-ranked minor road accident influencing factors into three groups. Fig. 7 represents the cluster-wise classification graph of all 82 minor factors influencing accident corresponding to their net flow value (phi). For a better descriptive analysis, every cluster in this graph is referred to as a severity level. Cluster 1 consists of the first 24 ranked minor factors. This means that cluster 1 has the highly severe road accident influencing factors, which caused the most fatalities, serious injuries, and mild injuries within Gujarat state. Similarly, the rest of the 30 and the last 28 ranked accident influencing factors were classified as moderately severe (cluster 2) and severe (cluster 3), respectively. This unique cluster-wise analysis is necessary to determine the severity level of each minor factor. Additional analysis is conducted to ascertain the characteristics and major factors associated with the dispersion of minor factors within clusters. Table 8 illustrates the ultimate severity rankings and distribution of each minor factor based on their characteristics and the major factors to which they belong.

**Table 8:** Final Cluster-wise distribution of minor factors according to characteristics and major road accident influencing factors for Gujarat state.

Major factor	Characteristics	Highly severe (Cluster 1)		Moderately severe (Cluster 2)		Severe (Cluster 3)	
		R^a^	Minor factor	R^a^	Minor factor	R^a^	Minor factor
Human factors	Gender	2	• Male (A75)	31	• Female (A76)		
	Driving role	10 8	• Drivers (A77) • Pedestrian & Passengers (A78)				
	No use of safety device	20	• Drivers not wearing helmets (A61)	42 54	• Passengers not wearing helmets (A62) • Drivers not wearing seatbelts (A63)	55	• Passengers not wearing seatbelts (A64)
	Traffic violation	1	• Over -Speeding (A56)			77 59 82 65	• Drunken driving/Consumption of alcohol & drug (A57) • Driving on wrong side (A58) • Jumping red light (A59) • Use of mobile phone (A60)
Environmental factors	Weather conditions	3	• Sunny/Clear (A1)	45	• Rainy (A2)	58 80	• Foggy & Misty (A3) • Hail/Sleet (A4)
	Time	22	• 18:00 to 21:00 (A69)	34 30 27 37 50	• 9:00 to 12:00 (A66) • 12:00 to 15:00 (A67) • 15:00 to 18:00 (A68) • 21:00 to 24:00 (A70) • 00:00 to 3:00 (A71)	56	• 3:00 to 6:00 (A72)
	Climatic seasons	21 12 16	• Summer (A79) • Monsoon (A80) • Winter (A82)	25	• Post-monsoon (A81)		
Road factors	Road type	11 19	• State Highways (A7) • National Highways (A6)			73	• Expressways (A5)
	Area type	6	• Open Area (A11)	29 48 41	• Residential (A8) • Institutional (A9) • Commercial (A10)		
	Locality	15 5	• Urban (A73) • Rural (A74)				
	Road feature	4	• Strait road (A12)	39	• Curved road (A13)	60 74 81 70 63	• Bridge (A14) • Culvert (A15), • Pot Holes (A16) • Steep Grade (A17) • Ongoing Road Works/Under Construction (A18)
	Junction type			52 51 44	• T Junction (A19) • Four Arm (A21) • Staggered (A22)	62 64	• Y Junction (A20) • Round About (A23)
	Traffic control	23	• Uncontrolled (A28)			72 69 76 78	• Traffic light Signal (A24) • Police controlled (A25) • Stop sign (A26) • Flashing signal/blinker (A27)
	Pedestrian infrastructure			32	• No pedestrian infrastructure (A32)	66 71 79	• Zebra crossing (A29) • Footpath (A31) • Foot Bridge/Subway (A30)
Vehicle factors	Vehicle type	9	• Two Wheelers (A35)	26 33 47 49	• Pedestrian (A33) • Cars, Taxis, Vans & LMV (A37) • Trucks/Lorries (A38) • Auto Rickshaws (A36)	67 68 75	• Buses (A39) • Bicycles (A34) • Other non - motorized vehicle (E -rickshaw etc.) (A40)
	Vehicle age	13 14	• Less than 5 years (A41) • 5–10 years (A42)	28 43	• 10.1–15 years (A43) • > 15 years (A44)		
	Vehicle loading	7 18	• Normally loaded (A45) • Empty (A47)	53	• Overloaded/hanging (A46)		
	Collision type	17 24	• Hit from back (A50) • Hit from side (A51)	35 36 40 46	• Hit & Run (A48) • Head on collision (A55) • Run off road (A52) • Vehicle Overturn (A54)	57 61	• Hit with fixed object (A53) • Hit with parked vehicle (A49)

a R: Rank.

Table 8 provides a clear depiction of the noteworthy observation that four out of the top ten minor factors influencing road accidents (i.e., A56, A75, A77, and A78) fall under the category of Human factors, which are classified as highly severe. This implies that human factors primarily have an influence on road accidents of a severe nature. Additional analysis indicated that the driving role and road localities are the distinguishing features that categorise each minor factor (i.e., A77, A78, A73, and A74) as highly severe. It is essential to recognise that characteristics such as driving duty, traffic offence, road type, and local traffic regulation do not fall under the category of moderately severe minor factors. Additionally, the characteristics of gender, driving role, climatic season, area type, locality, vehicle age, and vehicle load are the factors that are not classified as severe. This implies that these characteristics are contributing to the occurrence of road accidents of significant severity in the state of Gujarat. This final distribution of clusters offers a comprehensive and comparative analysis of the minor factors, characteristics, and major factors that contribute to road accidents in the state of Gujarat. The conventional statistical models or machine learning techniques used to analyse road accident severity do not readily offer this type of distribution.

The comprehensive assessment of 82 factors that contribute to road accidents will undoubtedly provide valuable insights for the development of road safety strategies and policies. For instance, in the event that the safety authority of Gujarat state is conducting an investigation into the factors causing to the occurrence of highly severe road accidents, the resulting severity ranking and distribution of factors and characteristics would yield a definitive response. Table 9 illustrates the five most significant factors and characteristics that have a substantial impact on road accidents in the state of Gujarat. Based on the presented data, it is advisable for safety authorities to consider implementing certain reforms. These reforms may include the enforcement of a comprehensive speed calming policy across the entire state, conducting road safety audits specifically targeting the straight roads that pass through rural areas within the state, and placing particular emphasis on addressing unsafe driving behaviours and road crossing patterns exhibited by male road users. Likewise, the prioritization of additional enforcement and policy reforms can be facilitated through an analysis of the findings of the current research. However, the successful implementation of these policy reforms, which is grounded on the current research findings, necessitates a systematic and coordinated approach. Given that two of the initial five factors that are highly severe, namely overspeeding and male gender, fall under the category of human factors, it necessitates additional time and effort to observe the significant alterations after any policy implementation. The updating process of human factors necessitates a significant amount of time. Moreover, third highly severe factor is sunny or clear weather conditions. Developing a policy reform for sunny weather poses challenges due to the inherently safe sight distance and visibility associated with such weather conditions. Hence, it is imperative to establish the correlation between this factor and other influential factors in road accidents in order to devise effective measures for ensuring road safety.

**Table 9:** Top five ranked - Highly severe factors and characterisctis influencing road accidents in Gujarat state.

Rank	Minor factors	Characteristics	Major factors
Highly severe			
1	Over -Speeding (A56)	Traffic violation	Human
2	Male (A75)	Gender	Human
3	Sunny/Clear (A1)	Weather condition	Environmental
4	Strait road (A12)	Road feature	Road
5	Rural (A74)	Locality	Road

## 5 Conclusion and recommendations

The present research is established with the aim of providing a novel application of the hybrid MCDM model to evaluate the severity of road accident influencing factors and provide analysis for the distribution of severity clusters, namely highly severe, moderately severe, and severe. To satisfy the aims, the research applies a hybrid MCDM model of the AHP and PROMETHEE and the k-mean clustering technique. The analysis has been done on the data of road accidents that resulted in fatalities, grievous injuries, and minor injuries within the Gujarat state of India. 40 experts have been contacted to establish the comparative weights of fatalities, grievous injuries, and minor injuries. Then, the resulted weights were included within the hybrid AHP-PROMETHEE decision-making approach to evaluate the severity of 82 minor factors influencing road accident severity. Before moving forward with the severity analysis and clustering, each of the 82 minor factors is categorized into 18 characteristics and 4 major factors for a more robust and clear analysis. The full potential of visual PROMETHEE software has been utilised to analyse the present research with accuracy and proper graphical representation.

The PROMETHEE study has been concluded with a detailed evaluation of 82 accident-influencing minor factors based on phi^−^, phi+, and phi values as per Fig. 3. The PROMETHEE I ranking based on phi^−^ and phi^+^ values as well as the PROMETHEE ranking based on phi values of 82 minor factors are totally similar. Additionally, it's interesting to note that there are no conflicts between the severity rankings of any minor factors provided by phi- and phi + values. This represents the robustness and clarity of the present research. Further, this research also represents the GAIA representation for every criteria, from which the severity influence of every factor can be easily established. Based on PROMETHEE I and II rankings and also with GAIA representation, minor factors such as over-speeding (A56), male (A75), sunny or clear weather (A1), straight road (A12), rural area (A74), open area (A11), normally loaded vehicle (A45), pedestrians and passengers (A78), two-wheelers (A35), and driver (A77) have been ranked among the top ten severe factors influencing road accidents. Further, the overall ranking results of 82 minor factors were classified into three groups using the k-mean clustering technique. Cluster 1 consists of the first 24 ranked minor factors with higher phi values. This means that cluster 1 has the most severe road accident influencing factors, which caused the most fatalities, serious injuries, and mild injuries within Gujarat state. Similarly, the rest of the 30 and the last 28 ranked accident influencing factors were classified as moderately severe (cluster 2) and severe (cluster 3), respectively. This severity distribution has been uniquely put together in Fig. 7 and Table 8. This one-of-a-kind severity distribution provides a clear severity distribution of any factor with details about the characteristics and major factors within which they belong.

Using existing accident records, this study uncovered a wealth of information about the characteristics and factors influencing the severity of road accidents. The result of the present hybrid AHP-PROMETHEE model establishes a policy-making basis to reduce road accidents by highlighting the severity of factors influencing road accidents within a particular area. Moreover, the enhanced approach incorporated an expert's input to establish the criteria weight of injury types. Further, the present approach effectively use the available road accident datasets without any pre-data preparation calculations. Therefore, one can compare the yearly severity performance of any particular road accident influencing factor and characteristic based on the available road accident datasets with the present approach. Overall, this research fills a gap in the available literature and have a clear advantage by providing a novel hybrid AHP-PROMETHEE to evaluate the severity of road accident influencing factors by considering fatalities, grievous injuries, and minor injuries and providing a clear severity classification of most factors influencing road accidents. Therefore, the adoption of this research may aid road accident experts and government officials in making more informed decisions. Using this finding, solutions for controlling the severity of accidents can be developed in multiple policy stages. Nonetheless, the quality of accessible road accident data has a significant impact on this study. Any miss reporting or over reporting of accident may result in minor to significant differences in the outcome of present research. Further, the effective interpretation of the results provided by the present research requires a proper understanding of every accident influencing factor and its characteristics. This understanding of individual factors and characteristics with the implementation of results provides a better policy base. Also, in future studies, more stakeholder groups can be involved in the evaluation process, since different stakeholder groups engagement can lead to increased advocacy for road safety measures. When citizens actively participate in discussions and decision-making related to road safety, they can influence policies that lead to safer road infrastructure, traffic management, and regulations.

## Funding statement

This work was supported by the Science Foundation of Ireland through the VOTE-TRA project (Funder reference: **22/NCF/DR/11309).**

## Data availability statement

Data will be made available on request.

## Additional information

No additional information is available for this paper.

## CRediT authorship contribution statement

**Priyank Trivedi:** Conceptualization, Data curation, Formal analysis, Methodology, Writing – original draft. **Jiten Shah:** Conceptualization, Software, Writing – original draft. **Sarbast Moslem:** Conceptualization, Methodology, Supervision, Writing – review & editing. **Francesco Pilla:** Supervision, Visualization, Writing – review & editing.

## Declaration of competing interest

The authors declare no conflict of interest.
